# Prospects and Research Trends of Tuberculosis in Iran: A Bibliometric Study With a Science Mapping Approach

**DOI:** 10.1155/ipid/7594073

**Published:** 2025-10-31

**Authors:** Sadeghali Azimi, Bahareh Bashardoust, Masoud Mohammadi

**Affiliations:** ^1^Department of Infectious Diseases, School of Medicine, Golestan University of Medical Sciences, Gorgan, Iran; ^2^Department of Medical Parasitology and Mycology, Faculty of Paramedicine, Golestan University of Medical Sciences, Gorgan, Iran; ^3^Department of Medical Information Sciences, School of Allied Medical Sciences, Golestan Research Center of Gastroenterology and Hepatology, Golestan University of Medical Sciences, Gorgan, Iran

**Keywords:** bibliometric, Iran, network visualization, research, science mapping, tuberculosis

## Abstract

**Background:**

Tuberculosis (TB), as one of the challenges of the health system in Iran, is of interest to Iranian researchers. It is necessary to present the research perspective of the research in this field to guide the future research. The aim of this research is bibliometric and citation study and providing a scientific map of TB research in Iran.

**Methods:**

This bibliometric and cross-sectional analyzes the research outputs of the subject area of TB indexed in the Scopus database up to 2024. SciMAT and VOSviewer software were used to visualize and predict the trends in research on the topic.

**Results:**

The most scientific productions occurred in the years 2020–2022 as well as 2016, and the research density was low in these years. Isoniazid and rifampicin were the most frequent topics in TB researches. In terms of average citations, diabetes mellitus had the most citations. Multidrug-resistant TB, diseases and complications, signs and symptoms, diagnosis and epidemiology of TB, and immunological and genetic studies related to TB are three research lines in TB research.

**Conclusion:**

TB research in Iran is expanding both quantitatively and in terms of research concepts. Thematic maps and strategic diagrams were presented. Paying attention to themes and strategic diagrams in the research decision-making of TB researchers in Iran in order to conduct effective research is helpful. Also, subject specialists in various fields of TB can obtain a specific research perspective based on the maps and indicators provided.

## 1. Introduction

Tuberculosis (TB), caused by bacteria from the *Mycobacterium tuberculosis* complex, is an age-old disease that has plagued humanity and continues to be a significant cause of mortality worldwide [1]. In 2021, approximately 10.6 million individuals worldwide contracted TB, representing a rate of 134 cases per 100,000 people. The majority of TB cases were concentrated in the South-East Asia (45%), Africa (23%), Western Pacific (18%), the Eastern Mediterranean (8.1%), the Americas (2.9%), and Europe (2.2%) regions according to the World Health Organization (WHO) [2]. The report by the WHO highlights that in Global Estimates of Mortality by Cause, TB is the first cause of death from an infectious agent and the 13th leading cause of death worldwide [2].

The results of a study on the incidence of pulmonary TB in Iran showed that the cumulative incidence was 18.52 per 100,000 population during 2014–2019. The incidence of smear-positive pulmonary TB declined from 23.84 per 100 000 population in 2014 to 15.02 in 2019 [3]. In another research based on the Global Burden of Disease Study 2019, it was stated that in the Middle East region, the highest prevalence rates of TB were in Egypt 28935.42, Lebanon 25899.77, and Iran 25364.71 [4]. Although the treatment for TB has been relatively successful, there has not been a significant decrease in TB incidence in Iran. The delay in diagnosing the disease, the high burden of TB among refugees, and the need for thorough contact investigation and prophylaxis are crucial factors in the TB control program in Iran [5].

In this regard, the importance of research in TB in national and international levels lies in its critical role in advancing our knowledge of the disease, creating innovative and effective treatments, improving diagnostic techniques, enhancing prevention efforts, addressing vulnerable populations, and ultimately, eradicating the illness [6]. A better understanding of research trends and scientific policies of researchers can create the best prospects for realizing the stated goals. Researchers can enhance their research endeavors in their specific field by acquiring a comprehensive comprehension of the scientific framework pertaining to their area of study. Consequently, it becomes imperative to visualize information and create a map that illustrates the scientific structure of the field. By constructing a scientific map based on the research outputs of a particular scientific domain, one can identify and depict the trends, research directions, and thematic clusters that have evolved over time [7–10]. Furthermore, this national-scale analysis of TB research production in Iran is particularly valuable for several compelling reasons. First, examining research trends at the national level helps identify critical gaps between scientific priorities and actual public health needs. Second, given Iran's unique healthcare infrastructure and specific challenges, our findings offer direct implications for domestic policy-making. Third, such national analyses serve as essential prerequisites for future international comparative studies. Finally, this approach enables the identification of strengths and weaknesses in local research efforts, which is crucial for strategic planning at Iranian research institutions.

Thus, aiming to create a well-documented research perspective in the field of TB research in Iran, this study examines the literature (research) published in this area and constructs its strategic map.

## 2. Material and Methods

Using the scientometric method, this cross-sectional study examined all documents on TB authored by Iranian researchers and indexed in the Scopus database up to 2024. The study applied a scientific mapping approach for data analysis. The reason for choosing the Scopus database is its international reputation, extensive and comprehensive research coverage, and its importance in the Iranian health research system. In Iran, the basis for evaluating researchers and institutions in terms of research is the documents indexed in this database and, therefore, choosing Scopus provides a more realistic perspective of the TB research system. A comprehensive methodology will be employed to retrieve the TB records in Iran. This entails conducting searches for all documents that bear any form of association with relevant terms. Consequently, the ensuing search plan will encompass the utilization of the following strategy: TITLE-ABS-KEY (Tuberclosis OR TB) AND AFFILICOUNTRY (Iran).

We used SciMAT v1.1.04 and VOSviewer Version 1.6.20 software to analyze the results. SciMAT (Science Mapping Analysis software Tool) is a powerful software tool that encompasses an array of methods, algorithms, and measures to facilitate each step of the general science mapping workflow. By utilizing SciMAT, users can conduct comprehensive studies based on multiple bibliometric networks, including coword, cocitation, author cocitation, journal cocitation, coauthor, bibliographic coupling, journal bibliographic coupling, and author bibliographic coupling. From preprocessing to result visualization, SciMAT offers a versatile platform for scientific analysis [11]. SciMAT, when utilized as a machine learning tool, was found to be a beneficial instrument for conducting science mapping analysis, as revealed by the study's findings [12]. VOSviewer, a software application designed for bibliometric visualization, was created by Nees Jan van Eck and Ludo Waltman at the Centre for Science and Technology Studies (CWTS) at Leiden University. Its diverse functionalities, such as specialized clustering algorithms and natural language processing capabilities, have contributed to the widespread adoption of VOSviewer within the Scientometrics field and beyond [13, 14].

The SciMAT-based strategic mapping of TB research entailed an examination of central bibliometric parameters, including author keywords alongside source and added terms, which are automatically derived from the titles of cited references [14]. The analytical configuration defined “Words” as the unit of analysis (encompassing author, source, and added roles), employed a co-occurrence network type, utilized the equivalence index for normalization and for measuring evolution and overlap, and applied the Centers simples clustering algorithm with a maximum and minimum cluster size of 100 and 2, respectively. To enable a granular and temporally relevant assessment, the investigation was segmented into five 2-year intervals: pre-2005, 2005–2010, 2010–2015, 2015–2020, and 2020–2024.

Utilizing VOSviewer software to draw a scientific map and conceptual illustration of the research required setting the keyword repetition threshold to a minimum of 100 repetitions. As a result, and by applying this condition, 119 keywords with at least 100 repetitions were included in the research. One of the most important issues in drawing scientific maps is cleaning words. In other words, very general words such as human, type of study (such as meta-analysis), and similar things that do not have an important meaning should be excluded from the study. In this research, after removing general words, 74 keywords were finally included in the study. Also, one of the challenges encountered during this phase pertained to the presence of diverse writing styles, including variations in singular and plural forms, as well as synonyms for the representation of lexical maps. In order to address this issue and ensure consistency in the depiction of concepts, the researchers devised a dedicated thesaurus specifically tailored for utilization in VOSviewer analysis. This represents a notable advantage of the VOSviewer software analysis, as it facilitates the unification of concepts and mitigates the potential dispersion of similar ideas.

## 3. Results

### 3.1. Baseline Characteristics of Studied Documents

As of June 13, 2024, a total of 4032 documents have been published by Iranian researchers in the Scopus database in the field of TB. This amount represents 0.07 percent of the participation of Iranian researchers in global production. Also, the trend of scientific productions in the field of TB by Iranian researchers shows that with a relatively gentle slope, the quantity of scientific productions has increased over time. The findings show that the most scientific productions occurred in the years 2020–2022 as well as 2016, and the research density was low in these years.

**Figure 1:**
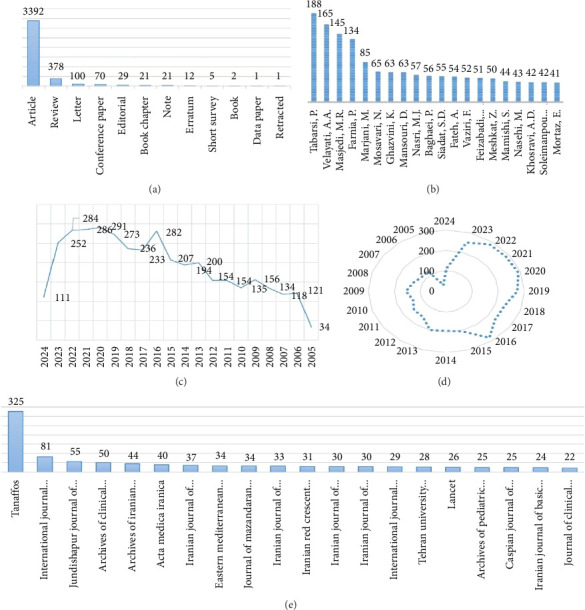
Baseline characteristics of studied documents. (a) Research studies in the field of TB in Iran based on the type of study. (b) The most prolific authors of TB field productions in Iran. (c) Number of research studies in the field of TB in Iran during 2005–2024. (d) Time density of production of TB in Iran. (e) The most prolific sources (journals) of TB field productions in Iran.

Core journals and authors play a central role in the production and development of knowledge. In this regard, the findings showed that the Tanaffos journal (ISSN: 0344-1735) was the most important journal in the publication of scientific productions in the field of TB in Iran (325 articles; 8.06 percent) and with a huge difference compared to other journals, it is the core journal in the field of TB research in Iran. The most productive author is Tabarsi Peyman (ORCID) with 188 articles (4.66% of all scientific productions) and he is the core author in this field. In terms of the type of studies, 3392 studies were original (84.12%) and 378 were reviews (9.37%). Other information is provided in Figure 1.

### 3.2. Map of Scientific Productions

**Figure 2:**
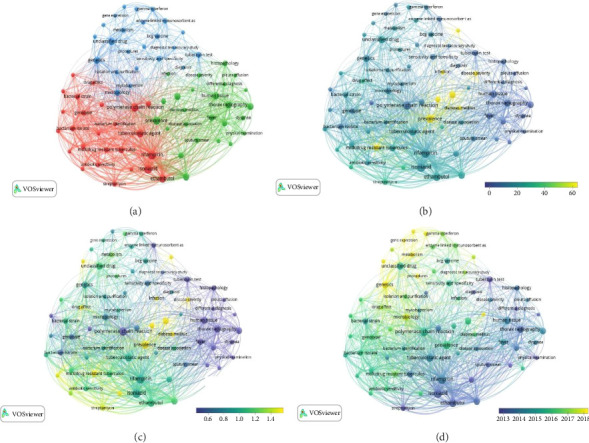
Visualization of the thematic map of research in the field of emergency medicine based on (a) co-occurrence analysis, (b) the average citation, (c) the average normal citation, and (d) the average publication year.

Table 1 and Figure 2 provided in this study illustrate the distribution of scientific research on TB through a co-occurrence analysis of words. The map reveals that the published documents are categorized into three distinct topic clusters. It is important to note that the placement of these topics within clusters is determined by their co-occurrence with one another, resulting in thematic themes. Clusters 1 and 2 consist of 25 nodes each, while Cluster 3 contains 23 nodes. It is worth mentioning that the research method employed in this study required a minimum of 100 repetitions for inclusion, which resulted in the exclusion of recent documents due to insufficient repetition. As time progresses, it is expected that the research landscape in this field will naturally undergo changes.

The findings showed that isoniazid and rifampicin were the most frequent topics with a frequency of 503 occurrences, followed by polymerase chain reaction with a frequency of 435 occurrences, and they were the focus and attention of TB researchers in Iran. All these topics are in Cluster 1.

In terms of citations, diabetes mellitus and infection had the most citations with an average of 281.44 and 177.15 citations, respectively. Both of these topics belong to Cluster 2. Also, in terms of the normal citation average, these two subjects were still at the top of the most cited subjects with average normal citation of 6.02 and 4.11. After these, incidence, human immunodeficiency virus (HIV) infection, disease severity, and prevalence with a normal citation average of more than 2 have been the most important topics in the field of TB research in Iran.

According to the average year of publication, the subjects of “treatment methods, gene expression and metabolism, complications, genetics, protein expression, and chemistry” attained the top ranking, with an average publication year of 2018. In other words, these topics have been the most up-to-date and latest topics of interest to TB researchers in Iran.

### 3.3. Characteristics of 3 Topic Clusters

#### 3.3.1. Cluster 1 (Yellow in Figure 2)

The subject of research in the first cluster, which is marked with yellow color in the map presented in Figure 2, was mainly related to multidrug-resistant (MDR) TB as well as genetic strains of *Mycobacterium tuberculosis*. This situation indicates the importance of drug resistance of *Mycobacterium tuberculosis* in current research studies. In this cluster, based on the number of links, the subjects of bacterium culture, ethambutol, isoniazid, polymerase chain reaction, rifampicin, and tuberculostatic agent have the most communication link and co-occurrence with 72 links with other subjects of this cluster. However, based on Total Link Strength (TLS), rifampicin has the highest association strength with TLS with a rate of 4081. TLS is a measure of the importance of a node or keyword within a network. The higher the TLS value, the more important the node is in the network. Based on the frequency of occurrence, the same topic with a frequency of 503 is at the top of the topics of this cluster. In terms of average citations, MDR TB had the most citations in Cluster 1 with an average of more than 68 citations. Based on the average number of normalized citations, drug effect has the highest value with 1.81. The normalized number of citations for a document is calculated by dividing the number of citations for that document by the average number of citations for all documents published in the same year and included in the dataset used by VOSviewer, as outlined in the VOSviewer manual [15, 16]. In terms of novelty index (average year of publication), the findings showed that the published documents on the topic of drug effect were mostly published in 2017, and it was the most up-to-date topic of interest to researchers in this cluster.

#### 3.3.2. Cluster 2 (Blue in Figure 2)

Research in Cluster 2 mainly revolves around diseases and complications related to TB, signs and symptoms of TB patients, diagnosis and treatment of TB, and epidemiology of this disease. According to the findings, the subjects of HIV infection, pyrazinamide, complication, thorax radiography, human tissue, fever, and coughing have the most subject links of this cluster with 72 links. In terms of TLS, pyrazinamide and thorax radiography are at the top of Cluster 2 topics with 2369 and 1883 connection power, respectively. In terms of occurrence, the topics of prevalence, thorax radiography, and pyrazinamide are the most important topics in TB research in Cluster 2 with frequencies of 390, 325, and 4230, respectively. In terms of average citations, diabetes mellitus had the most citations in this cluster with an average of 281.45 citations. Based on the average normal citation, diabetes mellitus still had the most citations in Cluster 2 with 6.03 citations. In terms of the novelty index (average year of publication), the findings showed that the documents published on complication with an average year of publication of 2018 were the most up-to-date topics of interest to researchers in this cluster.

#### 3.3.3. Cluster 3 (Green in Figure 2)

Cluster 3 mainly focuses on immunological and genetic studies related to TB. In this cluster, genetics, microbiology, isolation and purification, sensitivity and specificity, BCG vaccine, mycobacterium, and tuberculin test each have the most simultaneous links with 72 links, and genetics has the highest link strength with 1834. In this cluster, unclassified drug is the most frequent topic with 322 repetitions. But in terms of citations, the findings of our study show that latent TB has the most citations with an average of 76.48, followed by the concepts of immunology and unclassified drug with approximately 21 citations. In terms of average normalized citation, chemistry, latent TB, and metabolism had the most citations with 1.61, 1.58, and 1.26 citations, respectively. According to the findings, the subjects of procedures, gene expression, metabolism, genetics, protein expression, and chemistry had the most scientific production in 2018 and are the most up-to-date subjects of Cluster 3 of TB research in Iran.

### 3.4. Longitudinal Results

#### 3.4.1. Overlapping Map

**Figure 3:**
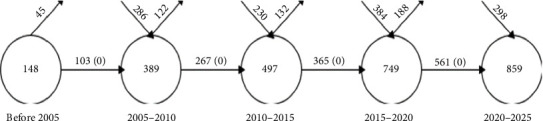
Overlap fractions (incoming and outcoming keywords between successive subperiods).

Figure 3 tracks the temporal dynamics of keywords through sequential time segments. The volume of keywords per segment is visualized by the size of each circle. Connectivity between segments is indicated by arrows, which quantify the keyword overlap (with a similarity index). The diagram also differentiates the inflow of novel keywords (upward arrows) from the outflow of discontinued keywords (downward arrows) between periods [14]. For instance, in the 5 subperiods analyzed (prior to 2005), there are a total of 148 keywords. Among these, 103 keywords continue into the next segment (2005–2010), while the remaining 45 keywords do not.

#### 3.4.2. Strategic Diagrams

**Figure 4:**
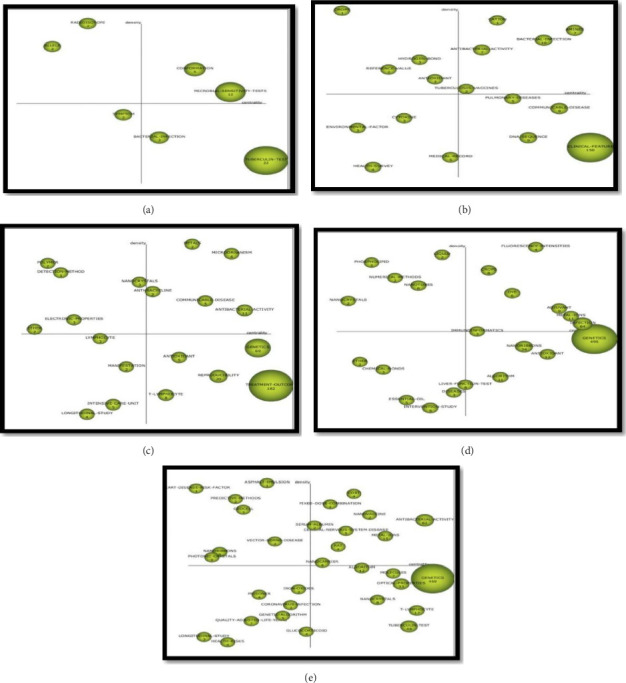
Strategic diagram based on number of published documents: (a) period before 2005, (b) period 2005–2010, (c) period 2010–2015, (d) period 2015–2020, (e) period 2020–2025.

**Figure 5:**
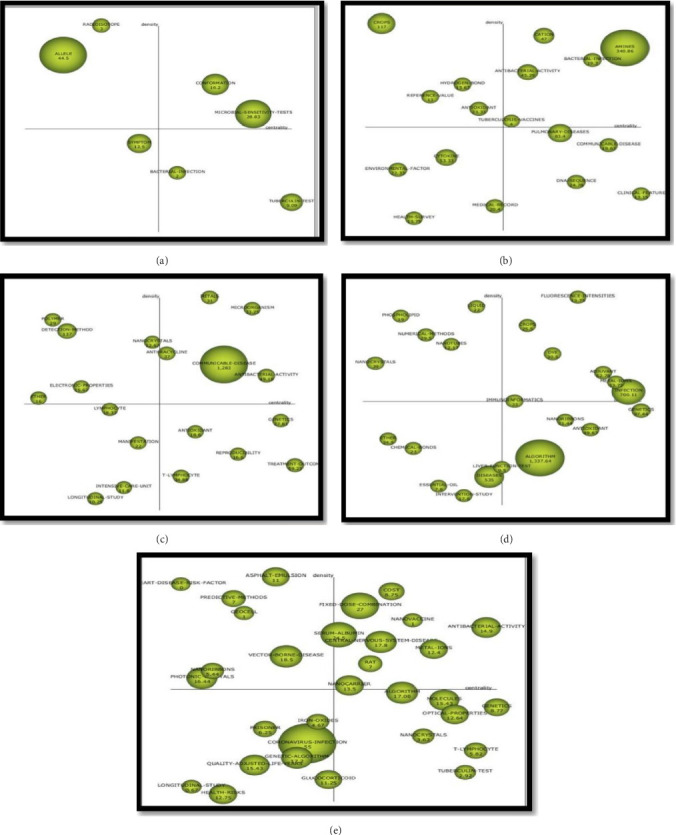
Strategic diagram based on the number of citations: (a) period before 2005, (b) period 2005–2010, (c) period 2010–2015, (d) period 2015–2020, (e) period 2020–2025.

One of the most important goals of this research is to identify and analyze thematic trends over historical periods, ultimately leading to the development of a strategic diagram that highlights critical areas for future investigation based on retrospective data. In this regard, TB research in Iran is divided into 5 time periods: before 2005, 2005–2010, 2010–2015, 2015–2020, and 2020–2025. First, subject clusters and Centrality, Density, Documents Count, Average Citations of each cluster in each time period were presented (Table 2). Then, based on these results, a strategic thematic diagram is presented based on two indicators: of the frequency of the number of published documents and the number of citations to the published sources in the thematic areas of the clusters indicated in Figures 4 and 5.

The interpretation of a strategic diagram is predicated on its foundational constructs, which are principally defined by two metrics: centrality and density. Centrality serves as a measure of a network's degree of interaction with other networks, quantifying the strength of its external ties. This metric functions as an indicator of a theme's relevance and influence within the broader development of the research domain. Conversely, density evaluates the network's internal cohesion by measuring the strength of interconnections among its constituent keywords. This measure is indicative of the theme's level of development and internal consistency [17, 18].

To further delineate the thematic composition of each sub-period, Table 1 summarizes key metrics—including centrality, density, publication volume, and average citation count—for every identified cluster. Analysis of these data confirms that clusters positioned in the upper-right quadrant of the strategic diagram function as core, motor themes. Characterized by strong centrality and high density, these themes are both highly developed and pivotal to the field's structural framework, maintaining robust external linkages to adjacent thematic areas. Conversely, themes in the upper-left quadrant, while internally well-developed (high density), exhibit limited external connections (low centrality), rendering them specialized yet peripheral to the broader discipline. Themes residing in the lower-left quadrant are identified as either emerging or declining, as they are underdeveloped, exhibiting both low density and low centrality. Finally, the lower-right quadrant contains themes of transversal importance to the field, signified by their high centrality, yet they remain underdeveloped, as indicated by their low density, suggesting they have not yet been fully realized [17].

In the period before 2005, Tuberculin-Test, Microbial-Sensitivity-Tests, and Conformation were the most central with 98.85, 44.58, and 24.58, respectively. Radioisotope, Bacterial-Infection, and Symptom clusters had the highest density with 72.18, 50, and 30.48, respectively. In other words, Tuberculin-Test, Microbial-Sensitivity-Tests, and Conformation clusters were the most important in the overall progress of TB research in Iran during this period, while Radioisotope, Coronally Bacterial-Infection, and Symptom clusters had the highest subject development within the cluster. Investigating the development of the TB research knowledge network in Iran since before 2005, the findings show that the highest research focus was on Tuberculin-Test, Microbial-Sensitivity-Tests, and Conformation clusters, which is consistent with the centrality index. But in terms of scholarly impact, an analysis of citation metrics reveals significant variation among the clusters. The Allele cluster emerges as a topic of significant importance, garnering the highest average citation count of 44.5. It is followed by the Microbial-Sensitivity-Tests (28.83), Conformation (16.2), and Symptom (12.5) clusters. The thematic strategic diagram for the pre-2005 period indicates that the Conformation and Microbial-Sensitivity-Tests clusters were positioned as motor themes. This placement signifies that they were not only internally well-developed but also played a vital, central role in the structure of TB research at the time. In contrast, the Radioisotope and Allele clusters were located in the highly developed and isolated quadrant, denoting a specialized but peripheral nature. Although central to TB research, the Tuberculin-Test and Bacterial-Infection clusters were classified as basic and transversal themes, suggesting they were important yet underdeveloped. Conversely, the Symptom cluster was identified as an emerging or declining theme. Notwithstanding their thematic positioning, the research strength and influence of these clusters vary considerably. This variance, quantified by the frequency of publications (document count) and citation volume, is illustrated in Figures 4 and 5.

For the timespan 2005–2010, the clusters Clinical-Feature, Amines, and Communicable-Disease exhibited the highest centrality values (334.16, 143.92, and 140.85, respectively), indicating their structural significance in the TB research network. In contrast, the clusters Crops, Cation, and Amines demonstrated the greatest density values (300.00, 257.14, and 178.47, respectively), reflecting strong internal development and thematic cohesion. The high centrality of Clinical-Feature, Amines, and Communicable-Disease underscores their pivotal role in the advancement of TB research, whereas the high-density clusters Crops and Cation represented areas of intensive, specialized knowledge development. Quantitatively, research emphasis aligned with centrality, focusing on these influential clusters; however, the Amines cluster also received the highest average citations (340.86), followed by Crops (117.00) and Pulmonary-Diseases (83.40). In the strategic diagram, motor themes—including Cation, Amines, Bacterial-Infection, Antibacterial-Activity, and TB-Vaccines—were identified as both well-developed and critical to the research domain. Conversely, clusters such as Crops, Hydrogen-Bond, Reference-Value, and Antioxidant occupied the highly developed and isolated quadrant, signaling specialized, niche relevance. Meanwhile, Clinical-Feature, DNA-Sequence, Communicable-Disease, and Pulmonary-Diseases were situated in the basic and transversal quadrant, denoting foundational but underdeveloped themes. Lastly, clusters including Cytokine, Environmental-Factor, Health-Survey, and Medical-Record were classified as emerging or declining, indicating peripheral or transitional status within the research landscape.

During the period from 2010 to 2015, the Treatment-Outcome, Genetics, and Antibacterial-Activity clusters recorded the highest centrality values—437.24, 340.25, and 203.4, respectively—reflecting their pronounced influence and integration within the broader research network. In contrast, the Metals, Microorganism, and Polymer clusters attained the highest density scores, measuring 252.08, 181.43, and 135.71, indicative of strong internal cohesion and thematic development. With regard to scholarly impact, the Communicable-Disease cluster emerged as particularly influential, amassing an average of 1283 citations. It was followed by the Detection-Method and Treatment-Outcome clusters, which received 137 and 59.23 citations on average, respectively. Thematic analysis situates the Metals, Microorganism, Communicable-Disease, and Antibacterial-Activity clusters within the motor quadrant, underscoring their role as central, well-developed themes. Clusters including Polymers, Detection-Method, Nanocrystals, and Electronic-Properties reside in the highly developed yet isolated quadrant, characteristic of specialized but peripherally connected themes. Conversely, the Genetics, Treatment-Outcome, Antioxidant, and T-Lymphocyte clusters occupy the basic and transversal quadrant, suggesting thematic importance within TB research that remains underdeveloped. Lastly, the cluster comprising Lymphocyte, Manifestation, Intensive-Care-Unit, and Longitudinal-Study is positioned in the emerging or declining quadrant, reflecting its nascent or diminishing role within the field.

From 2015 to 2020, the Genetics, Infection, and Metal-Ions clusters have demonstrated the highest centrality values, with 822.72, 309.22, and 193.83, respectively. An analysis of cluster metrics reveals distinct thematic strengths. The Fluorescence-Intensities, Liquid, and Phospholipid clusters demonstrated the highest internal cohesion, with density values of 97.77, 87.5, and 87.5, respectively, underscoring their well-developed nature. In terms of scholarly impact, the Algorithm cluster emerged as the most influential with an average of 1337.64 citations, followed by the Infection (700.11) and Diseases (535) clusters. The strategic positioning of these clusters is further elucidated by the thematic map. Clusters such as Fluorescence-Intensities, Dye, Crops, Adjuvant, Metal-Ions, and Infection are situated in the motor quadrant, signifying their dual role as both highly developed and central to the research field. Conversely, the Phospholipid, Liquid, Nanocrystals, Nanotubes, and Numerical-Methods clusters occupy the highly developed yet isolated quadrant, indicating advanced internal development but limited external connections. In contrast, the Algorithm, Nanoribbons, Antioxidant, and Genetics clusters are positioned in the basic and transversal quadrant. This placement denotes their high importance (centrality) to the field of TB research but suggests they are not yet fully developed, highlighting a critical need for further investigation to realize their potential. Finally, the Essential-Oil, Intervention-Study, and Chemical-Bonds clusters reside in the emerging or declining quadrant, characterized by low development and marginality.

In the period from 2020 to 2025, Genetics, Antibacterial-Activity, T-Lymphocyte, and Tuberculin-Test clusters have the highest Centrality with 1222.05, 679.11, 227.44, and 196, respectively. Heart-Disease-Risk-Factor (113.33), Cost (85.42), and Antibacterial-Activity (68.38) clusters have the highest density. In terms of citations, the findings show that the Coronavirus-Infection cluster with an average of 55 citations was one of the most important topics in this field, followed by Fixed-Dose-Combination cluster with 27 citations. Based on the thematic strategic diagram, Cost, Fixed-Dose-Combination, Nanovaccine, Nanocarrier, Antibacterial-Activity, Metal-Ions, Central-Nervous-System-Disease, and Serum-Albumin clusters are located in the engine quadrant. The strategic diagram positions several clusters within the highly developed and isolated quadrant, namely, Asphalt-Emulsion, Heart-Disease-Risk-Factor, Predictive-Methods, Geocell, Photonic-Crystals, and Nanoribbons. This placement indicates these themes possess strong internal cohesion but exhibit limited external connections to other areas of the field. Conversely, the Genetics, T-Lymphocyte, Tuberculin-Test, and Nanocrystals clusters are situated in the basic and transversal quadrant. Despite their recognized importance to TB research, this classification suggests they are not yet fully developed, highlighting a significant potential for future investigation. Finally, clusters including Glucocorticoid, Coronavirus-Infection, and Genetic-Algorithm occupy the emerging or declining quadrant, denoting that they are presently both peripheral and underdeveloped within the research landscape.

## 4. Discussion

This study presents the first comprehensive bibliometric analysis of TB research in Iran, employing scientometric tools to map its evolution, thematic clusters, and research gaps. Our findings highlight three dominant research lines—MDR TB, clinical and diagnostic studies, and immunological/genetic research—which align with global priorities but reveal critical gaps specific to the Iranian context.

The most frequently studied topics in Iranian TB research were isoniazid and rifampicin, reflecting their central role as first-line treatments worldwide [19, 20]. However, the near absence of pharmacovigilance studies on these drugs is striking, particularly given their well-documented risks of hepatotoxicity and neurotoxicity [21–25]. This gap poses a significant challenge to patient safety, especially in populations with comorbidities or genetic predispositions to adverse drug reactions.

Our cluster analysis identified three major research themes. The first centers on MDR TB, with a strong focus on antibiotic resistance mechanisms, such as *rpoB* mutations [26], and susceptibility testing. This aligns with global concerns about the rising threat of drug-resistant TB [27, 28]. The second theme encompasses clinical and diagnostic research, including epidemiology, comorbidities like diabetes and HIV coinfections [29], and diagnostic tools such as thorax radiography. Notably, studies linking diabetes to TB have received high citation impact, consistent with meta-analyses confirming diabetes as a significant risk factor for TB [30–33]. The third theme revolves around immunological and genetic studies, including research on BCG vaccine efficacy and biomarkers for latent TB. While this area has grown in recent years, it remains less densely explored compared with drug-focused research.

A closer look at publication trends reveals a surge in genetic and biochemical studies post-2018, likely driven by advances in molecular epidemiology and the increasing availability of genomic tools. These studies, which explore *Mycobacterium tuberculosis* polymorphisms and host–pathogen interactions, represent a promising shift toward precision medicine in TB research [34–39].

Despite these advances, several gaps persist. Pharmacovigilance and second-line therapies are notably underrepresented in Iranian TB research. Given the country's high burden of MDR TB, the lack of studies on second-line drugs, such as bedaquiline [40], or strategies to manage adverse drug reactions is concerning. This gap may stem from resource allocation favoring first-line treatments or limited infrastructure for pharmacovigilance.

The COVID-19 pandemic further disrupted TB research in Iran, with a 56% decline in publications between 2022 and 2024. This decline likely reflects the reallocation of healthcare resources to pandemic response and the diagnostic challenges posed by overlapping respiratory symptoms between COVID-19 and TB [41, 42].

Our analysis revealed a significant decline in tuberculin skin test (TST) research from 2005 to 2020, coinciding with global adoption of interferon-gamma release assays (IGRAs) following 2005 CDC guidelines [43]. The introduction of QuantiFERON-TB Gold (and its subsequent generations) shifted diagnostic standards worldwide, with over 125 million tests distributed globally [44].

In post-2020, Iranian research shows renewed interest in TST alongside continued IGRA studies, reflecting practical challenges of IGRA implementation, cost considerations for mass screening, and need for context-specific diagnostic approaches [45]. This dual focus suggests Iranian researchers are evaluating both tests' complementary roles rather than viewing them as mutually exclusive alternatives. The findings highlight the importance of developing localized diagnostic guidelines that consider Iran's healthcare infrastructure and epidemiological context. Future research should address cost-benefit analyses and quality improvement for both diagnostic methods in Iranian settings.

This study has several limitations. The reliance on the Scopus database may exclude regional journals or non-English publications, potentially underestimating the full scope of Iranian TB research. The citation lag for recent high-impact articles could also skew interpretations of emerging trends. Furthermore, thematic overlap between clusters, such as genetics and immunology, may not be fully captured by the clustering algorithms used.

To address these gaps and build on current research, we recommend several steps. First, integrating pharmacovigilance systems into TB control programs is essential to monitor and mitigate adverse drug reactions, aligning with the WHO safety guidelines. Second, expanding research on second-line therapies is critical to address the needs of patients with drug-resistant TB or those who cannot tolerate first-line regimens. Finally, leveraging interdisciplinary approaches, such as genomics and artificial intelligence, could enhance Iran's capacity to tackle MDR TB and align with global innovations in TB research.

## 5. Conclusion

In conclusion, this study provides a detailed map of TB research in Iran, highlighting its strengths, gaps, and opportunities for future work. By addressing these gaps and embracing emerging technologies, Iran can strengthen its TB research landscape and contribute more effectively to global efforts to combat this persistent public health challenge.

## Figures and Tables

**Table 1:** Thematic clusters of TB research in Iran and detailed information on nodes based on the documents indexed in the Scopus database.

Node (concept)	Cluster	Weight (links)	Weight (total link strength)	Weight (occurrences)	Score (avg. pub. year)	Score (avg. citations)	Score (avg. norm. citations)
Amikacin	1	68	994	104	2016.058	33.9808	1.6749
Antibiotic resistance	1	69	1648	197	2015.579	19.9898	1.115
Antibiotic sensitivity	1	71	1563	171	2016.023	17.6433	1.0842
Antitubercular agents	1	71	1952	246	2013.862	22.1585	1.1076
Bacterial gene	1	64	836	106	2016.057	10.7736	0.4881
Bacterial strain	1	64	1378	202	2014.852	19.0644	1.0398
Bacterium culture	1	72	1383	224	2013.821	12.3036	0.6918
Bacterium identification	1	67	837	124	2014.895	17.4274	0.9674
Bacterium isolate	1	65	1605	213	2015.789	10.9014	0.6044
Bacterium isolation	1	66	821	108	2014.176	18.0185	0.8885
DNA extraction	1	66	765	132	2016.629	5.4697	0.3438
Drug effect	1	69	786	106	2017.226	31.5377	1.807
Ethambutol	1	72	3184	369	2013.352	22.206	1.0669
Gene mutation	1	65	841	106	2015.943	13.8302	0.7409
Genotype	1	66	1085	189	2016.164	12.6455	0.6354
Isoniazid	1	72	4037	503	2013.356	18.2048	0.9482
Microbial sensitivity tests	1	65	1265	133	2015.399	18.1128	1.0239
Minimum inhibitory concentration	1	66	759	117	2016.231	19.7692	1.1475
Multidrug resistance	1	67	1059	120	2015.692	23.75	1.4416
Multidrug-resistant tuberculosis	1	71	1626	203	2016.695	68.2906	1.7472
Polymerase chain reaction	1	72	2451	435	2015.361	9.3195	0.5086
Rifampicin	1	72	4081	503	2014.036	17.2227	0.9337
Streptomycin	1	70	1555	166	2011.651	29.5723	1.4123
Tuberculosis, multidrug resistant	1	67	1122	118	2015.941	30.0763	1.3228
Tuberculostatic agent	1	72	2550	399	2014.341	20.5639	0.979
Bronchoscopy	2	54	453	101	2013.317	5.7723	0.3105
Complication	2	72	505	102	2018.147	17.1667	0.8059
Computer-assisted tomography	2	67	1441	267	2014.277	4.573	0.3073
Coughing	2	72	1284	195	2013.723	5.9179	0.3085
Diabetes mellitus	2	68	477	130	2016.139	281.4462	6.0279
Differential diagnosis	2	58	591	114	2012.904	8.0789	0.4697
Disease association	2	70	567	126	2014.008	18.1746	1.0846
Disease severity	2	70	545	130	2014.108	127.2231	2.4469
Dyspnea	2	66	783	151	2014.291	5.2649	0.3029
Epidemiology	2	60	360	107	2014.692	29.3832	0.7993
Erythrocyte sedimentation rate	2	64	751	112	2015.75	5.6161	0.3102
Fever	2	72	1573	247	2014.551	6.0769	0.4126
Histopathology	2	66	995	189	2013.714	7.5608	0.4007
Human immunodeficiency virus infection	2	72	1015	261	2015.996	110.6897	2.5617
Human tissue	2	72	1639	288	2014.184	6.1319	0.3527
Incidence	2	69	642	188	2015.495	157.1436	3.266
Infection	2	71	351	108	2015.963	177.1574	4.1118
Physical examination	2	60	738	119	2013.025	6.1261	0.3338
Pleura effusion	2	61	534	104	2014.096	6.4135	0.4258
Prevalence	2	71	1659	390	2015.933	96.4718	2.1126
Pyrazinamide	2	72	2369	291	2013.495	24.1478	1.1483
Sputum smear	2	64	876	163	2013.319	12.5521	0.6577
Thorax radiography	2	72	1883	325	2013.48	7.0123	0.4046
Treatment outcome	2	69	1176	210	2013.105	17.681	0.9575
Weight reduction	2	64	733	104	2011.673	9.0096	0.3993
BCG vaccine	3	72	951	189	2014.91	16.1693	0.9065
Chemistry	3	56	473	119	2018.008	19.6891	1.6185
Diagnosis	3	65	280	110	2007.764	12.8455	0.797
Diagnostic test accuracy study	3	69	471	100	2017.06	9.99	0.5886
Enzyme linked immunosorbent assay	3	71	865	179	2016.62	8.6592	0.5174
*Escherichia coli*	3	66	463	114	2017.29	15.6842	1.0226
Gamma interferon	3	68	752	147	2016.939	13.8367	0.9831
Gene expression	3	65	489	102	2018.471	12.3529	1.0115
Genetics	3	72	1834	293	2018.137	15.3242	0.8918
Human cell	3	69	583	126	2016.421	12.7937	0.8282
Immune response	3	65	575	128	2017.789	18.6094	1.1688
Immunology	3	64	630	137	2016.496	21.6569	0.8946
Isolation and purification	3	72	939	125	2016.432	15.568	0.835
Latent tuberculosis	3	60	436	107	2017.393	76.4766	1.5833
Metabolism	3	70	768	149	2018.336	19.6577	1.2637
Microbiology	3	72	1663	233	2017.588	14.3648	0.725
*Mycobacterium*	3	72	646	110	2014.327	16.4273	1.003
*Mycobacterium bovis*	3	67	553	111	2013.631	14.1441	0.7401
Procedures	3	70	601	129	2018.589	19.5504	1.1596
Protein expression	3	67	527	106	2018.038	12.2453	0.7711
Sensitivity and specificity	3	72	1013	227	2015.458	11.2159	0.6875
Tuberculin test	3	72	1098	219	2012.58	7.2603	0.3783
Unclassified drug	3	71	1251	322	2015.354	21.2516	1.1099

**Table 2:** Subject clusters and features of each cluster in TB research in Iran, based on 5 time periods of study.

Subperiod	Cluster name	Centrality	Density	Documents count	Average citations
Before 2005	Tuberculin-Test	98.85	20.98	22	9.09
	Microbial-Sensitivity-Tests	44.58	21.42	12	28.83
	Conformation	24.58	23.7	5	16.2
	Radioisotope	3.75	72.18	2	3
	Symptom	20.32	30.48	2	12.5
	Bacterial-Infection	21.42	50	3	2
	Allele	0	20.98	2	44.5

2005–2010	Clinical-Feature	334.16	32.65	150	13.15
	Amines	143.92	178.47	7	340.86
	Bacterial-Infection	107.09	103.91	10	19.3
	Communicable-Disease	140.85	39.79	9	19.67
	Antibacterial-Activity	49.86	82.02	7	45.29
	DNA-Sequence	71.92	35.81	9	24.78
	Antioxidant	39.89	56.79	3	23.33
	Pulmonary-Diseases	59.25	47.88	5	83.4
	Medical-Record	41.43	18.18	5	20.4
	Crops	0	300	1	117
	Cation	54.17	257.14	1	47
	Hydrogen-Bond	33.92	78.57	3	13.67
	Health-Survey	10.22	12.43	4	13.75
	Tuberculosis-Vaccines	43.05	56.25	2	4
	Reference-Value	11.67	64.58	2	13
	Environmental-Factor	0.81	36	3	22.33
	Cytokine	31.42	37.78	3	53.33

2010–2015	Treatment-Outcome	437.24	20.01	182	59.23
	Genetics	340.25	47.38	69	27.81
	Antibacterial-Activity	203.4	85.09	11	49.18
	Reproducibility	138.31	37.6	20	16.6
	Microorganism	158.46	181.43	4	33.25
	Communicable-Disease	122.19	98.59	5	1283
	Antioxidant	77.54	43.99	5	18.6
	Metals	86.33	252.08	2	31
	Electronic-Properties	12.64	78.19	3	15.67
	T-lymphocyte	72.6	18.75	8	34.88
	Nanocrystals	33.67	102.22	3	12.67
	Intensive-Care-Unit	25.35	15.71	5	11.8
	Polymer	8.06	135.71	2	29
	Lymphocyte	18.9	59.18	3	18.33
	Longitudinal-Study	12.79	13.61	4	10.25
	Anthracycline	34.75	100	2	37
	Detection-Method	10.58	125	1	137
	Ether	2.5	75	2	16
	Manifestation	31.96	40	2	22

2015–2020	Genetics	822.72	33.38	495	97.44
	Infection	309.22	37.73	64	700.11
	Nanoribbons	94.49	33.27	36	21.44
	Metal-Ions	193.83	43.04	11	33.73
	Antioxidant	123.34	31.66	12	49.67
	Adjuvant	158.2	48.48	9	52.78
	Dye	84.72	59.94	6	30.5
	Algorithm	80.75	23.67	11	1337.64
	Fluorescence-Intensities	111.18	97.77	4	13.75
	Crops	59.92	84.15	8	25.5
	Nanotubes	24.56	61.06	6	12.17
	Liver-Function-Test	44.24	21.25	6	9.5
	Intervention-Study	25.29	7.66	5	17.8
	Numerical-Methods	23.75	80.95	2	29.5
	Phospholipid	18.98	87.5	2	19
	Nanocrystals	5.27	52.78	2	39
	Immunoinformatics	50.22	35.19	3	33
	Liquid	30.09	87.5	2	22
	Essential-Oil	24.39	13.43	5	7.8
	Chemical-Bonds	21.55	25.28	3	23
	Diseases	31	20.83	3	535
	Ether	14.01	26.11	2	24.5

2020–2025	Genetics	1222.05	35.19	469	8.77
	Antibacterial-Activity	679.11	35.19	62	14.9
	Tuberculin-Test	196	68.38	48	2.92
	Optical-Properties	180.07	16.39	33	12.64
	T-lymphocyte	227.44	32.15	17	5.82
	Metal-Ions	138.5	27.65	15	12.4
	Molecules	157.62	50.95	7	15.43
	Algorithm	81.89	38	12	17.08
	Nanocrystals	133.59	41.28	8	3.62
	Nanoribbons	15.53	31.41	9	5.44
	Cost	57.37	46.38	4	8.75
	Photonic-Crystals	11	85.42	9	16.44
	Central-Nervous-System-Disease	55.01	45.37	5	17.8
	Rat	50.25	54.96	4	7
	Coronavirus-Infection	26.07	47.78	3	55
	Genetic-Algorithm	24.59	29.43	5	17.2
	Nanovaccine	117.54	20.14	2	1
	Geocell	17.21	68.45	3	1
	Nanocarrier	42.41	74.4	4	13.5
	Prisoner	18.81	42.5	4	6.25
	Fixed-Dose-Combination	44.89	31.9	2	27
	Serum-Albumin	39.69	75	2	24.5
	Iron-Oxides	31.97	58.33	3	4.67
	Quality-Adjusted-Life-Years	17.83	31.94	7	15.43
	Asphalt-Emulsion	20	19.31	1	11
	Heart-Disease-Risk-Factor	3.91	125	1	0
	Predictive-Methods	16.67	113.33	2	7
	Vector-Borne-Disease	23.43	75	2	18.5
	Glucocorticoid	38.76	50	4	11.25
	Longitudinal-Study	4.4	12.25	3	0.67
	Health-Risks	15.6	11.5	4	12.75

## Data Availability

All the relevant data supporting the findings of this study are available from the corresponding author upon reasonable request.
